# Emergency in obese patients: a reply to SOBA UK

**DOI:** 10.1186/s44158-022-00040-z

**Published:** 2022-03-17

**Authors:** Ida Di Giacinto, Martina Guarnera, Clelia Esposito, Stefano Falcetta, Gerardo Cortese, Giuseppe Pascarella, Massimiliano Sorbello, Rita Cataldo

**Affiliations:** 1 Unit of Anesthesia and Intensive Care, Mazzoni Hospital, Ascoli Piceno, Italy; 2 Department of Anesthesia and Intensive Care, Azienda Ospedaliero-Universitaria Sant’Orsola-Malpighi - Alma Mater Studiorum, Bologna, Italy; 3 Department of Anesthesia and Intensive Care, Ospedali dei Colli, Napoli, Italy; 4 Department of Anesthesia and Intensive Care, Clinica di Anestesia e Rianimazione Ospedali Riuniti, Ancona, Italy; 5 Department of Anesthesia and Intensive Care, AOU Città della salute e della scienza, Torino, Italy; 6 Department of Anesthesia and Intensive Care, Università Campus Bio-Medico, Roma, Italy; 7 Department of Anesthesia and Intensive Care, AOU Policlinico San Marco University Hospital, Catania, Italy

**Keywords:** Resuscitation, Obesity, Emergency, Cardiac arrest, Trauma

## Abstract

Emergency settings in obese people require tailored multidisciplinary protocols and pathways to manage these complex patients. For this reason, we would like to foresee a proficient cooperation with the UK Society for Obesity and Bariatric Anaesthesia (SOBA) and other societies: obesity is a worldwide problem, and an international and multidisciplinary cooperation is desirable, if not needed. As demonstrated for bariatric surgery, a standardizing anesthesiologic and critical approach and an experienced multidisciplinary staff, trained and equipped to manage obese patients, are related to better outcomes. Similarly, as recently pointed out for airway management safety, we believe that the presence of an *obese lead* should be a desirable goal to reach in the next future, especially when thinking of emergency situations and the need for resuscitation of obese patients. A worldwide problem calls for worldwide cooperation.

Editor,

We read with interest and really appreciated the comments by Dr Mckechnie and colleagues [1] on our recent review [2]. We believe that the most important issue raised by our colleagues’ letter is the ubiquitous attention to obesity, and its implications for perioperative and emergency care. We would like to develop collaborative working between the UK Society for Obesity and Bariatric Anaesthesia (SOBA) and other societies: obesity is a worldwide problem, and an international and multidisciplinary cooperation is desirable, if not needed.

Tailored multidisciplinary protocols and pathways to manage these complex patients are required. We believe that dedicated teams should be adequately trained for both technical and non-technical skills for emergency and perioperative management of this *heavily frail* population, including resuscitation, advanced procedures, and transport. Education, training, simulation and organization are the only tools we believe appropriate to face the challenge of “*Globesity*”, a pandemic without vaccine. Not by chance, the interest of the Italian Society of Anesthesia, Analgesia, Resuscitation and Intensive Care (SIAARTI) regarding patients with obesity has widely increased in the recent years, leading us to publish consensus, good clinical practices, and itinerant training programs among clinicians [3, 4].

As demonstrated for bariatric surgery, standardizing the anaesthetic and critical care approach, alongside an experienced multidisciplinary staff, trained and equipped to manage patients with obesity**,** is related to better outcomes [5, 6]. Similarly, as recently pointed out for airway management safety [7], we believe that the presence of a *lead for obesity*, aiming to ensure indications and supply of dedicated obesity equipment, diffusion of knowledge including implementation of practice guidelines, organizational and educational programs, should be a desirable goal to reach in the near future. This is especially important when thinking of emergency situations and the need for resuscitation of patients with obesity [8].

Despite evidence showing the need for closing these specific gaps, and the further lessons from COVID-19 pandemic, which heavily affected the population living with obesity on one hand [9], and demonstrated the importance of structured and teamwork approach on the other [10], no specific recommendations have been published yet regarding life support for patients with obesity.

This may depend on the lack of high-quality evidence, so we believe that the first common effort of international societies should be high-quality research to build such evidence and to develop shared consensus documents.

A worldwide problem calls for worldwide cooperation.

## Data Availability

Not applicable

## References

[1] McKechnie A, Pengelly L, Cousins J (2021) on behalf of SU. SOBA UK response to “emergencies in obese patients: a narrative review”. Journal of Anesthesia, Analgesia and. Crit Care 1(1):25. 10.1186/s44158-021-00027-2 10.1186/s44158-021-00027-2 PMC10245399 37386533

[2] Di Giacinto I, Guarnera M, Esposito C et al (2021) Emergencies in obese patients: a narrative review. Journal of Anesthesia, Analgesia and. Crit Care 1(1):13. 10.1186/s44158-021-00019-2 10.1186/s44158-021-00019-2 PMC8590435 37386567

[3] Petrini F, Di Giacinto I, Cataldo R et al (2016) Perioperative and periprocedural airway management and respiratory safety for the obese patient: 2016 Siaarti Consensus. Minerva Anestesiol 82(12):1314–1335 27759743

[4] Marinari G, Folletto M, C N et al (2022) Enhanced recovery after bariatric surgery: an Italian consensus statement. Surg Endosc Accepted 10.1007/s00464-022-09498-y PMC9485178 35953683

[5] Geubbels N, Evren I, Acherman YIZ, Bruin SC, Laar AWJM, Hoen MB, Brauw LM (2019) Randomized clinical trial of an enhanced recovery after surgery programme versus conventional care in laparoscopic roux-en-y gastric bypass surgery. BJS Open 3(3):274–281. 10.1002/bjs5.50143 31183442 10.1002/bjs5.50143 PMC6551390

[6] Ruiz-Tovar J, Garcia A, Ferrigni C, Gonzalez J, Castellon C, Duran M (2019) Impact of implementation of an enhanced recovery after surgery (eras) program in laparoscopic roux-en-y gastric bypass: a prospective randomized clinical trial. Surg Obes Relat Dis 15(2):228–235. 10.1016/j.soard.2018.11.002 30606469 10.1016/j.soard.2018.11.002

[7] Baker PA, Behringer EC, Feinleib J, Foley LJ, Mosier J, Roth P, Wali A, O’Sullivan EP (2022) Formation of an Airway Lead Network: an essential patient safety initiative. Br J Anaesth 128(2):225–229. 10.1016/j.bja.2021.11.013 34893313 10.1016/j.bja.2021.11.013

[8] Gupta T, Kolte D, Mohananey D, Khera S, Goel K, Mondal P, Aronow WS, Jain D, Cooper HA, Iwai S, Frishman WH, Bhatt DL, Fonarow GC, Panza JA (2016) Relation of obesity to survival after in-hospital cardiac arrest. Am J Cardiol 118(5):662–667. 10.1016/j.amjcard.2016.06.019 27381664 10.1016/j.amjcard.2016.06.019

[9] Cai Z, Yang Y, Zhang J (2021) Obesity is associated with severe disease and mortality in patients with coronavirus disease 2019 (COVID-19): a meta-analysis. BMC Public Health 21(1):1505. 10.1186/s12889-021-11546-6 34348687 10.1186/s12889-021-11546-6 PMC8334342

[10] Sorbello M, Morello G, Pintaudi S, Cataldo R (2020) COVID-19: Intubation Kit, Intubation Team, or Intubation Spots? Anesth Analg 131(2):e128–e130. 10.1213/ANE.0000000000004970 32404591 10.1213/ANE.0000000000004970

